# The perceived impact of the COVID-19 pandemic on medical students’ future careers

**DOI:** 10.12688/f1000research.73249.1

**Published:** 2021-11-26

**Authors:** Heba Mahjoub, Chirag Vasavda, Amanda Bertram, Ashwini Davison, Stephen Sozio

**Affiliations:** 1 Department of Medicine, Johns Hopkins University School of Medicine, Baltimore, Maryland, 21205, USA; 2 The Solomon H. Snyder Department of Neuroscience, Johns Hopkins University School of Medicine, Baltimore, Maryland, 21205, USA; 3 Department of Epidemiology, Johns Hopkins Bloomberg School of Public Health, Baltimore, Maryland, 21205, USA

**Keywords:** medical education, COVID-19 pandemic, medical students, career planning

## Abstract

**Background**: The COVID-19 pandemic disrupted medical education on multiple levels, and medical students have been forced to adjust to distance learning, altered clinical opportunities, and standardized testing inconsistencies. We sought to identify the effects of these dramatic deviations on medical students’ career plans.

**Methods**: We conducted a cross-sectional online survey of medical students between July 13, 2020, and September 9, 2020 in order to assess the implications of the COVID-19 pandemic on students’ career decisions. Descriptive statistics were calculated for all variables.

**Results**: Of the 585 eligible medical students, we had a final sample of 76 responses (n=76) (13% response rate). Students felt neutral regarding having more time to explore research projects (Mean ± SD; 3.06 ± 1.18) and hobbies (3.43 ± 1.28). Most survey respondents somewhat disagreed that they considered quitting medical school during the pandemic (1.55 ± 1.10). Students somewhat agreed that they view the field of medicine more positively since the onset of the COVID-19 pandemic (3.60 ± 1.09). Respondents somewhat agreed that they would be unable to explore other specialties and find their best fit (3.55 ± 1.32). We found that the minority (4/66, 6%) of students had considered changing their specialty. Students felt neutral in terms of their Step 1 (3.25 ± 1.05) or Step 2 (2.81 ± 1.02) score deterring them from future career opportunities.

**Conclusions**:  Most medical students have experienced barriers in their career pathway as a direct cause of COVID-19 restrictions on medical education, including the ability to explore different specialties to discover their best fit or find a chance to network with mentors. However, despite these obstacles, most students remain committed to medicine.

## Introduction

The Coronavirus disease-2019 (COVID-19) pandemic created unforeseen obstacles for humankind across the globe. Tasks that were once mundane have now become difficult or unsafe. Importantly, medical students have been uniquely affected. For many months, the Association of American Medical Colleges (AAMC) advised medical schools across the United States to pause in-person education in order to slow the spread of the virus. ^1^ Most medical schools transitioned pre-clinical curricula to an online format, embracing learning from a distance. Unfortunately, clinical years, where medical students serve directly on medical teams rather than in a classroom, were more difficult to transfer online. ^2^ To further complicate the situation, mandatory standardized tests such as USMLE Step 1 and Step 2 were postponed or cancelled for many students. ^3^ Many medical students have also been unable to continue their scientific research and volunteer opportunities in the community. Such experiences are crucial in career development as they help shape students’ interests.

One learning theory that forms the theoretical framework for this study is connectivism, which emphasizes the importance of technology and socialization for building an educational framework. ^4^ It especially highlights that learning is a continuous process that requires nurturing connections between study areas. ^5^ Interestingly, this theory supports both virtual learning and in-person learning, muddying the potential outcomes of a transition from clinical rotations to e-learning. While different students have been affected by COVID-19 in various ways, ^6^ the aggregate impact of the pandemic will resonate throughout both medicine and society for years to come. Hence, we sought to study the implications of the COVID-19 pandemic on students' career decisions. In particular, we aimed to investigate the impact of COVID-19 on medical students’ perspectives towards medicine. We explored whether the pandemic may have altered students’ professional and personal goals, such as whether interests in specialties had changed, whether they were questioning medicine as a career, and whether they felt well-educated on pandemics and infectious diseases. We also aimed to determine students’ reactions to changes in licensing exams and how this uncertainty might influence their careers.

## Methods

### Ethical approval and consent

This research was acknowledged and exempted by the Johns Hopkins Institutional Review Board (IRB00248820). Prior to survey administration, participants received emails stating that their voluntary participation in the survey represented their informed written consent to participate.

### Research design

We conducted a cross-sectional survey between July 13, 2020, and September 9, 2020. Requests for participation were sent out to Johns Hopkins students via Qualtrics ^®^ version XM using individual email links, followed by three reminder emails every 2-3 weeks. Medical students currently enrolled in Johns Hopkins University School of Medicine pursuing a Doctor of Medicine (MD), MD/Doctor of Philosophy (PhD), MD/ Master of Public Health (MPH), MD/Master of Business Administration (MBA) degree upon graduation were included. The survey items were developed to cover topics ranging from physical and emotional well-being during the pandemic, as well as perceptions of the effects on career development (Underlying data). ^7^ Emotional well-being was ascertained according to validated assessment. ^8^ Respondents rated statements according to a 5-point Likert scale, with a score of 1 representing “strongly disagree”, 2 “somewhat disagree”, 3 “neutral”, 4 “somewhat agree”, and 5 “strongly agree”. Descriptive statistics were calculated for all variables using Qualtrics ^®^ version XM. version XM.

All individuals surveyed were medical students who were affected by changes to their medical education due to the COVID-19 pandemic. The study is sensitive to selection bias for students that responded to and completed the survey, potentially limiting the generalizability of our findings from all students. Additionally, our survey only reached medical students at a single institution and thus may not reflect the experience of students at other institutions. The study size was determined as the total number of medical students enrolled and eligible to receive the survey by email. All data were included in the analyses.

## Results

Of the 585 eligible medical students, we received 83 total responses. Seven responses were excluded because only the first question was answered, leaving a final sample of 76 responses (n = 76) (13% response rate). Most respondents were women (43/76, 57%), and the average age was 25.68 (± 2.45 standard deviation (SD)). In this sample, 51% (39/76, 51%) were white, followed by 29% (22/76, 29%) Asian or Pacific Islander, 8% (6/76, 8%) multi-racial, and 8% (6/76, 8%) Black or African American. Many students had received prior degrees including two with PhDs (2/76, 2%), three with Master of Public Health (MPH)s (3/76, 4%), three with Master of Science (MS)s (3/76, 4%), and one with a Master of Arts (MA) (1/76, 1%). No students (0/71, 0%) were offered an early graduation option by their medical school at the height of the pandemic ( Table 1).

**Table 1.** Characteristics of respondents to the COVID-19 medical student survey.

Variable	N (%)
Degree received upon graduation	
MD	52 (68)
MD/PhD	23 (30)
MD/MPH	1 (1.0)
Gender	
Woman	43 (57)
Man	29 (38)
Genderqueer	1 (1.3)
Gender non-binary	2 (2.6)
Prefer not to respond	1 (1.3)
Age	
Mean 25.68 ± 2.45 SD	
Hispanic, Latina/o, or of Spanish origin	
Yes	4 (5)
No	72 (95)
Race	
White	39 (51)
Asian or Pacific Islander	22 (29)
Black or African American	6 (8)
South Asian	1 (1)
Multi-racial	6 (8)
Prefer not to respond	2 (2.4)
Previous graduate degrees received	
PhD	2 (2)
MPH	3 (4)
MS	3 (4)
MA	1 (1)
Prefer not to respond	1 (1)
None	66 (87)
Early graduation option given by medical school	
Yes	0 (0)
No	71 (100)
N/A	5
Highest year of medical school completed excluding MD/PhD	
MS1	22 (41.5)
MS2	12 (22.6)
MS3	15 (28.3)
MS4	3 (5.7)
MPH	1 (2)
Highest year of medical school completed excluding non-MD/PhD	
GS1	2 (9)
GS2	1 (4)
GS3	5 (22)
GS4	0 (0)
GS5+	3 (13)
MS1	8 (35)
MS2	1 (4)
MS3	3 (13)

A series of COVID-19 related questions were included in the survey ( Table 2) ( Underlying data). ^7^ When asked the question ‘Are there currently restrictions in the place where you live?’, 50% (50/100, 50%) of students responded ‘My town/city is in the process of easing restrictions’, as well as 46 students (46/100, 46%) who responded ‘Social isolation is recommended by state or local authorities. Of those who responded, 25% (25/71, 35%) had friends or family members who had been diagnosed with COVID-19, although none (0/71, 0%) were themselves diagnosed with COVID-19. In the overall cohort, most respondents (37/71, 52%) were leaving their home four or more times per week for work, shopping, or other reasons.

**Table 2.** COVID-19 restrictions reported by medical students during the COVID-19 pandemic.

Variable	N (%)
Are there currently restrictions in the place where you live?	
There is a stay-at-home order given by state or local authorities.	2 (2)
Social isolation is recommended by state or local authorities.	46 (46)
I am being quarantined because of potential exposure.	1 (1)
My town/city is in the process of easing restrictions.	50 (50)
There are no restrictions now, but there were restrictions in the past.	1 (1)
Do you live with someone at high risk of morbidity/mortality from COVID-19? (immunocompromised, elderly, pre-existing conditions)	
Yes	11 (16)
No	60 (85)
N/A	5
Have any of your friends/family been diagnosed with COVID-19?	
Yes	25 (35)
No	46 (65)
N/A	5
Have you been diagnosed with COVID-19?	
Yes	0 (0)
No	71 (100)
N/A	5
Have you had symptoms of COVID-19, but never received testing to confirm?	
Yes	6 (8.4)
No	65 (92)
N/A	5
How often do you leave home for work, shopping, or any other reason?	
0 times per week	4 (5.6)
1 time per week	10 (14)
2 times per week	11 (15)
3 times per week	9 (12)
4+ times per week	37 (52)
N/A	5

Several survey questions ( Table 3) covered student well-being. On average, students somewhat agreed with currently feeling that they have a definite role among family and friends (Mean ± SD; 3.89 ± 1.00), that they are useful to family and friends (3.80 ± 1.13), that they are being listened to (3.87 ± 0.92), and that they know what is going on with their family and friends (4.18 ± 0.86). Students felt neutral regarding having more time to explore research projects (3.06 ± 1.18) and hobbies (3.43 ± 1.28), as well as more time to spend with friends or family members (3.34 ± 1.37). Table 3 shows additional responses regarding student well-being. In a section of our survey that permitted free text, students shared their difficult experiences. An example of one experience was ( Extended data) ^9^:

**Table 3.** Self-perceived personal well-being, prior education, perceived obligations, and career planning experienced by medical students during the COVID-19 pandemic.

Statement	Mean ± SD	Variance
I currently feel that I have a definite role among my family and friends.	3.89 ± 1.00	1.00
I currently feel I am useful to my family and friends.	3.80 ± 1.13	1.29
I currently feel I am being listened to.	3.87 ± 0.92	0.85
I currently feel I know what is going on with my family and friends.	4.18 ± 0.86	0.74
I currently feel I can talk about my deepest problems.	4.11 ± 0.91	0.83
I have more time to take care of my personal wellness.	3.57 ± 1.19	1.41
I have more time to explore research projects.	3.06 ± 1.18	1.38
I have more time to explore hobbies.	3.43 ± 1.28	1.63
I have more time to spend with friends or family members.	3.34 ± 1.37	1.89
Prior to the COVID-19 pandemic, my medical education adequately informed me about epidemics.	3.49 ± 1.08	1.16
Prior to the COVID-19 pandemic, my medical education adequately informed me about infection prevention and control.	3.65 ± 1.05	1.11
My confidence in the healthcare system has been shaken.	3.34 ± 1.37	1.87
My confidence in my medical school’s administration has been shaken.	3.12 ± 1.33	1.78
I have considered quitting medical school during the pandemic.	1.55 ± 1.10	1.20
I believe that it is my duty to inform society about COVID-19 prevention and control.	3.96 ± 0.92	0.85
I view the field of medicine more positively since the onset of COVID-19 pandemic.	3.60 ± 1.09	1.20
I believe it is my responsibility to volunteer during this pandemic.	3.37 ± 1.12	1.25
I am unable to explore other specialties and find my best fit.	3.55 ± 1.32	1.73
I am unable to network with potential mentors.	3.79 ± 1.15	1.33
I feel less comfortable forming relationships with faculty members.	3.11 ± 1.28	1.65
I feel less comfortable asking for letters of recommendation.	2.97 ± 1.22	1.48
I feel like I will not be competitive for my desired field.	2.71 ± 1.08	1.17
I am unable to start new research projects.	3.37 ± 1.32	1.75
I am unable to complete old research projects.	3.03 ± 1.33	1.77
I am worried that canceled away rotations will limit my career trajectory.	2.89 ± 1.22	1.48
I am more worried about performing well on graded clerkships than before.	3.13 ± 1.37	1.88
I am worried that I won’t have the opportunity to complete sub-internships and/or advanced electives at my institution.	3.18 ± 1.34	1.79

*“I was recently diagnosed with major clinical depression. While being quarantined and having to contend with the ever more present issue of police brutality against BIPOC individuals [Black, Indigenous and People of Color] and systemic racism, I often feel exhausted, unmotivated, useless, and general sadness which makes it extremely difficult to wake up and go about my days.”*

Students were also asked about their medical education prior to COVID-19 and their perceived obligations ( Table 3). When questioned about the adequacy of their medical education regarding epidemics, they felt neutral (3.49 ± 1.08). Students felt neutral regarding their confidence in the healthcare system (3.34 ± 1.37) and their medical school’s administration (3.12 ± 1.33). Most survey respondents somewhat disagreed that they considered quitting medical school during the pandemic (1.55 ± 1.10). The students somewhat agreed that they view the field of medicine more positively since the onset of the COVID-19 pandemic (3.60 ± 1.09) and that it is their responsibility to volunteer during the pandemic (3.37 ± 1.12). Table 3 indicates additional responses covering medical education prior to COVID-19 and perceived obligations.

There were multiple survey questions regarding obstacles to mentorship and residency preparation ( Table 3). Respondents somewhat agreed that they would be unable to explore other specialties and find their best fit (3.55 ± 1.32), as well as being unable to network with potential mentors (3.79 ± 1.15). Students were neutral regarding the level of comfort forming relationships with faculty members (3.11 ± 1.28), asking for letters of recommendation (2.97 ± 1.22), and level of competitiveness for their desired field (2.71 ± 1.08). In terms of their career, students were neutral that losing the ability to participate in away rotations would limit their career trajectory (2.89 ± 1.22), that they would not perform as well on graded clerkships as before (3.13 ± 1.37), and that they would not have the opportunity to complete sub-internships or advanced electives at their institution (3.18 ± 1.34).

**Figure 1.**
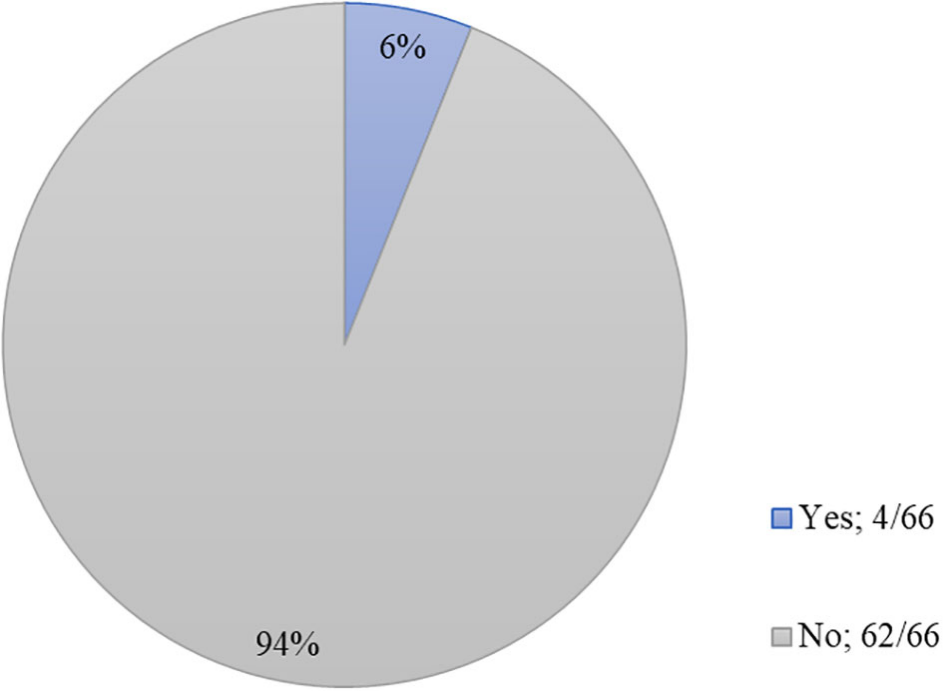
Have you considered changing your preferred specialty choice since the onset of the COVID-19 pandemic?

We were also curious as to whether the pandemic had caused students to switch their preferred specialty and the effects of the pandemic on the standardized exams, Step 1 and Step 2. We found that the minority (4/66, 6%) of students had considered changing their specialty. For those who had considered changing, these swaps were from emergency medicine to neurosurgery, otolaryngology to internal medicine, neurosurgery to internal medicine, and internal medicine to anesthesiology ( Figure 1). Six students (6/66, 9%) absolutely believed that the COVID-19 pandemic would negatively impact their current or future Step 1 score, while 13 (13/66, 19.6%) did not believe that it would. The majority (37/66, 56.0%) of students did not believe the pandemic had negatively affected their current or future Step 2 score. Neither group felt strongly that their Step 1 (3.25 ± 1.05) or Step 2 (2.81 ± 1.02) score would deter them from future career opportunities ( Table 4). One student shared their experience with the testing sites during the pandemic ( Extended data) ^9^:

**Table 4.** Pandemic effects on licensing exams experienced by medical students.

Variable	N (%)
Do you think the COVID-19 pandemic has negatively impacted your current/future Step 1 score?	
Yes	6 (9)
No	13 (19.6)
Maybe	19 (28.8)
I took Step 1 before the COVID-19 pandemic.	28 (42.4)
If yes or maybe, the effect on my Step 1 score deters me from future career opportunities:	
Mean 3.25 ± 1.05	
Do you think the COVID-19 pandemic has negatively impacted your current/future Step 2 score?	
Yes	2 (3.0)
No	37 (56.0)
Maybe	26 (39.4)
I took Step 2 before the COVID-19 pandemic.	1 (1.5)
If yes or maybe, the effect on my Step 2 score deters me from future career opportunities:	
Mean 2.81 ± 1.02

*“I think the biggest frustration for a lot of people has been uncertainty of their test date, as testing appointments continued to get cancelled last minute. It's incredibly frustrating and exhausting to prepare for months for a specific day, just to find out in the days before that you'll have to change your date and continue studying.”*

## Discussion/conclusion

In this cross-sectional survey, we explored the important topic of how the COVID-19 pandemic has influenced medical students’ professional goals. We analyzed 76 responses from diverse medical students with various educational backgrounds. Most students perceive barriers in their career pathway, including the ability to explore different specialties to discover their best fit or find a chance to network with mentors. However, despite these obstacles, most students feel strongly to continue in medicine on the same pathway that they began.

We found that many students lived in a town or city that was in the process of easing restrictions (50/100, 50%), but that social isolation was still recommended by state or local authorities (46/100, 46%). Some students (11/71, 16%) were even living with high-risk populations. These factors led medical schools to pause in-person learning. Though transitioning classes to an online format might seem effective, the impact of isolated learning has already taken an effect on students. Even when learning has continued in a digital, socially distanced format, studies have shown that students are experiencing disheartening decreases in overall work performance and emotional detachment. ^10^ This is concerning, considering the highest quality of patient care comes from doctors working effectively as teams. ^11^

Interestingly, students in our study did not feel they had more or less time to spend on research projects (3.06 ± 1.18), on hobbies (3.43 ± 1.28), or with friends and family (3.34 ± 1.37). Although students did not feel an increase or decrease in time spent on research projects, they did feel strongly that other opportunities for career preparation were stunted. Respondents somewhat agreed that they would have difficulty exploring other specialties to find their best fit (3.55 ± 1.32), as well as being unable to network with potential mentors (3.79 ± 1.15). Overall, however, respondents were neutral when questioned about their competitiveness for their desired field (2.71 ± 1.08). Further, students might have felt that their Step 1 or Step 2 scores were affected by the pandemic, but students did not feel strongly that these scores would deter them from future career opportunities. These findings indicate that while certain experiences have been limited by the pandemic, students generally see themselves on the same path that they initially envisioned. We found that only the minority (4/66, 6%) of students had considered changing their specialty. This is less than a previous study’s finding that one-fifth of medical students would change their choice of specialty. ^12^ This study, by Byrnes et al., involved a larger sample size including students at various institutions, but the findings showed that region of the US did not alter whether the pandemic affected students’ specialty choices. Furthermore, our study found that students felt neutral (2.97 ± 1.22) in asking for letters of recommendation, whereas the respondents in the Byrnes et al. study posed that as their main cause of specialty change.

In addition, most survey respondents somewhat disagreed that they considered quitting medical school during the pandemic (1.55 ± 1.10). Coupled with the data that these students view the field of medicine more positively since the onset of the COVID-19 pandemic (3.60 ± 1.09), this suggests a resilient attitude by the respondents. This highlights why certain medical schools across the country offered their students a chance to graduate early and join the frontlines tackling the COVID-19 pandemic; medical students want to practice medicine, and they understand the need for dedicated providers. ^13^ In addition, many previous studies have provided suggestions for engaging medical students throughout this pandemic, ^14^^,^^15^ and it is reassuring to see medical students taking a stand for their communities and their education by staying engaged. ^16^^,^^17^

Though our sample population offers a unique perspective from each student, our study comes with certain limitations. Our response rate of 13% may reflect some non-responder bias as well as survey fatigue, as our survey was sent at a time when many students were experiencing distressing times. A more robust sample size might provide additional and more diverse viewpoints. Additionally, all respondents came from a single institution in the mid-Atlantic region. Students at institutions in regions that experienced very different case numbers, such as in the Midwest, South, or West, might have different experiences that alter their career trajectories. Lastly, there were a handful of respondents who left certain questions unanswered, leaving us with missing information for that student.

Despite the challenges in distance-learning and career preparation that many are now experiencing, our survey study finds that students still have their minds set on achieving their pre-determined goals. We are hopeful that the hardships brought on by the COVID-19 pandemic have strengthened the next generation of physicians and that future patients benefit from their dedication.

## Data availability

### Underlying data

Figshare: The Perceived Impact of the COVID-19 Pandemic on Medical Students’ Future Careers.

DOI: https://doi.org/10.6084/m9.figshare.16879090.v1 ^7^

This project contains the following underlying data:

• Data file: Study survey questionnaire.

### Extended data

Repository: The Perceived Impact of the COVID-19 Pandemic on Medical Students’ Future Careers.

DOI: https://doi.org/10.6084/m9.figshare.16879087.v1 ^9^

This project contains the following underlying data:

• Data file: Student comments regarding step exams and other concerns.

Data are available under the terms of the Creative Commons Zero “No rights reserved” data waiver (CC0 1.0 Public domain dedication).

## Author contributions

All authors were involved in the conceptualization, investigation, methodology, project administration, supervision, validation, visualization, writing of the original draft, and reviewing and editing the subsequent drafts. HM and CV performed data curation and formal analysis. SMS was involved in funding acquisition, resources, and software.
