# Supplementary material for: The perceived impact of the COVID-19 pandemic on medical students’ future careers
Source: F1000Res. 2023 Feb 24;10:1211. Originally published 2021 Nov 26. [Version 2] doi: 10.12688/f1000research.73249.2 (PMC9989541; doi:10.12688/f1000research.73249.2)
Supplement: Peer review history

# Peer review: Reviewer response for version 2

**DOI:** 10.5256/f1000research.144443.r164542

**Published:** 2023-03-06

**Authors:** Jason Yuen

**Affiliations:** 1 Department of Neurologic Surgery, Mayo Clinic, Rochester, MN, USA

I think the revision has satisfied my concerns.

Is the work clearly and accurately presented and does it cite the current literature?

Yes

If applicable, is the statistical analysis and its interpretation appropriate?

Partly

Are all the source data underlying the results available to ensure full reproducibility?

Partly

Is the study design appropriate and is the work technically sound?

Yes

Are the conclusions drawn adequately supported by the results?

Partly

Are sufficient details of methods and analysis provided to allow replication by others?

Yes

Reviewer Expertise:

My main clinical interests are in neurosurgery, particularly neuromodulation. In addition, I am interested in medical education and have completed a postgraduate certificate in medical education. I have also taught, examined and mentored medical students on multiple occasions.

I confirm that I have read this submission and believe that I have an appropriate level of expertise to confirm that it is of an acceptable scientific standard.

# Peer review: Reviewer response for version 1

**DOI:** 10.5256/f1000research.76890.r146011

**Published:** 2022-08-23

**Authors:** Aikaterini Dedeilia

**Affiliations:** 1 Postdoctoral Research Fellow, Surgical Oncology Research Laboratories, Department of Surgery, Massachusetts General Hospital, Boston, MA, USA; 2 Research Fellow in Surgery, Harvard Medical School, Boston, MA, USA

The present manuscript is a cross-sectional survey among medical students, on the effect of COVID-19 on their education and future career plans. The study is comprehensible and consistent, and adequately presents all the required information about the study process. I believe that it can be indexed, provided that the authors consider the following suggestions/comments and make any appropriate adjustments:

1. In the introduction, although it provides an adequate background for the study, I would suggest that more citations are added in the sentences that lack one and present an argument that needs verification (e.g.: "Tasks that were once mundane have now become difficult or unsafe. Importantly, medical students have been uniquely affected." or "Many medical students have...in the community.")
2. In methods, the study design is adequately presented for any reader to understand. I would suggest that the authors add at the end of the first paragraph that continuous variables are presented as a mean followed by a standard deviation (mean ± SD), and that categorical variables as presented as n (%). Also, the phrase "version XM" is mentioned twice by accident in the same paragraph. Lastly, although it is highly important and proper of the authors to write the selection bias statement in the end of the methods, I would suggest that this limitation be included along with the rest of the limitations at the end of the discussion, along with the second limitation (about the single institution) that is repeated in the discussion.
3. The results are clear, legible, easily understandable and reproducible. I have no further comments or further improvements to make.
4. The discussion is related to the results, and the authors appropriately comment on the effect of COVID-19 on the career plans of medical students. I would suggest that more citations from similar studies are added in the discussion, in order to compare this study to others, or that some systemetic reviews that have studied this exact effect are added so that they further support the evidence from this study.
5. Last but not least, if possible from the format of the journal guidelines, I would suggest that the last paragraph be presented as a separate conclusion (with its own subtitle), instead of as a part of a common "discussion/conclusion" section. 

In conclusion, I have no further concerns or ethical reservations with regards to the present manuscript. I believe that it is a clear, reproducible, and technically sound study, that with some minor revisions can be indexed and add significant information to the literature.

Is the work clearly and accurately presented and does it cite the current literature?

Yes

If applicable, is the statistical analysis and its interpretation appropriate?

Yes

Are all the source data underlying the results available to ensure full reproducibility?

Yes

Is the study design appropriate and is the work technically sound?

Yes

Are the conclusions drawn adequately supported by the results?

Yes

Are sufficient details of methods and analysis provided to allow replication by others?

Yes

Reviewer Expertise:

medical education, surgical education, surgical oncology, general surgery, pediatric surgery

I confirm that I have read this submission and believe that I have an appropriate level of expertise to confirm that it is of an acceptable scientific standard.

# Response

**Published:** 2023-02-16

**Authors:** Heba Mahjoub

**Affiliations:** Johns Hopkins University School of Medicine, USA

**Reviewer 2, Aikaterini Dedeilia, Postdoctoral Research Fellow, Surgical Oncology Research Laboratories, Department of Surgery, Massachusetts General Hospital, Boston, MA, USA;  Research Fellow in Surgery, Harvard Medical School, Boston, MA, USA:**

**Approved**

**Reviewer's comment:**

 The present manuscript is a cross-sectional survey among medical students, on the effect of COVID-19 on their education and future career plans. The study is comprehensible and consistent, and adequately presents all the required information about the study process. I believe that it can be indexed, provided that the authors consider the following suggestions/comments and make any appropriate adjustments:

 In the introduction, although it provides an adequate background for the study, I would suggest that more citations are added in the sentences that lack one and present an argument that needs verification (e.g.: "Tasks that were once mundane have now become difficult or unsafe. Importantly, medical students have been uniquely affected." or "Many medical students have...in the community.")

**Authors response: **Thank you for your encouraging feedback regarding the importance of these findings. We are eager to improve the work with your suggestions. We have added the following citations in our introduction section:

*Tasks that were once mundane, such as scheduling test dates, have now become difficult or unsafe. ^a^*

*Many medical students have also been unable to continue their scientific research and volunteer opportunities in the community. ^b^*

*a. Price, S. (2020). Testing Boundaries COVID-19 made the USMLE, Clerkships a Moving Target for Med Students. Tex. Med. 34:116.*

*b. Pop AI, Mirel S, Florea M, Lotrean LM. The Impact of the COVID-19 Pandemic on Research and Volunteering Activities among Medical Students: A Cross-Sectional Study among Romanian and International Students from One Medical Faculty from Romania. Int J Environ Res Public Health. 2022;19(12):7477. doi:10.3390/ijerph19127477*

**Reviewer's comment:**

 In methods, the study design is adequately presented for any reader to understand. I would suggest that the authors add at the end of the first paragraph that continuous variables are presented as a mean followed by a standard deviation (mean ± SD), and that categorical variables as presented as n (%). Also, the phrase "version XM" is mentioned twice by accident in the same paragraph. Lastly, although it is highly important and proper of the authors to write the selection bias statement in the end of the methods, I would suggest that this limitation be included along with the rest of the limitations at the end of the discussion, along with the second limitation (about the single institution) that is repeated in the discussion.

**Authors response: **We appreciate your recommendation to include further description of how we represented our variables. We also have now removed the 2 ^nd^ “version XM” that was erroneously included previously. Last, we removed the selection bias statement from the methods and inserted it in the discussion section along with other limitations. We have adjusted the methods as follows:

*“Descriptive statistics were calculated for all variables using Qualtrics ^®^ version XM. Continuous variables are presented as a mean followed by a standard deviation (Mean ± SD), and categorical variables are presented as N (%).”*

 The following line was inserted into the discussion section:

*“The study is sensitive to selection bias for students that responded to and completed the survey, potentially limiting the generalizability of our findings from all students.”*

**Reviewer's comment:**

 The results are clear, legible, easily understandable and reproducible. I have no further comments or further improvements to make.

**Authors response: **Thank you for this positive feedback regarding the results!

**Reviewer's comment:**

 The discussion is related to the results, and the authors appropriately comment on the effect of COVID-19 on the career plans of medical students. I would suggest that more citations from similar studies are added in the discussion, in order to compare this study to others, or that some systemetic reviews that have studied this exact effect are added so that they further support the evidence from this study.

**Authors response: **Thank you for the suggestion to include more citations in the discussion section. We have now included the following line and citations to support our findings:

*These findings align with several other studies conducted nationally and internationally. ^a,b,c^*

*a. Alsoufi A, Alsuyihili A, Msherghi A, et al. Impact of the COVID-19 pandemic on medical education: Medical students’ knowledge, attitudes, and practices regarding electronic learning. PLoS One. 2020;15(11):e0242905. doi:10.1371/journal.pone.0242905*

*b. Nicholas Pari Tekkis, Damir Rafi, Sam Brown, et al. (2022) The impact of the COVID-19 pandemic on UK medical education. A nationwide student survey, Medical*

*Teacher, 44:5, 574-575, DOI: 10.1080/0142159X.2021.1962835*

*c. The perceived impact of the Covid-19 pandemic on medical student education and training – an international survey. BMC Med Educ. 2021;21:566. doi:10.1186/s12909-021-02983-3*

**Reviewer's comment:**

 Last but not least, if possible from the format of the journal guidelines, I would suggest that the last paragraph be presented as a separate conclusion (with its own subtitle), instead of as a part of a common "discussion/conclusion" section.

 In conclusion, I have no further concerns or ethical reservations with regards to the present manuscript. I believe that it is a clear, reproducible, and technically sound study, that with some minor revisions can be indexed and add significant information to the literature.

**Authors response: **Thank you for this suggestion, we have now separated the conclusion to stand on its own with its own subtitle. We appreciate your diligent work reviewing this study, and for your suggestions for improvement! We believe the edits we have made with your recommendations have made this a stronger manuscript.

# Peer review: Reviewer response for version 1

**DOI:** 10.5256/f1000research.76890.r118871

**Published:** 2022-01-10

**Authors:** Jason Yuen

**Affiliations:** 1 Department of Neurologic Surgery, Mayo Clinic, Rochester, MN, USA

The authors of this paper conducted a single-institution, cross-sectional study of the perspectives of medical students on how the COVID pandemic impacted their education, well-being and future career. In particular, the authors found that, based on answers in the form of Likert scales from an online survey, medical students encountered difficulties in exploring different specialties. Yet, they continued to view medicine as a positive career choice.

Pros

 How COVID-19 impacts medical education remains highly topical given the evolving nature of the pandemic, although numerous articles on a similar topic have been published for the past 2 years. The questions of the survey were well-designed to capture the numerous ways COVID might affect a medical student.

Cons

 The study is heavily limited by the low response rate (13%). Therefore, the results may be confounded by selection bias, which the authors have rightly acknowledged.

 Furthermore, a significant proportion of the samples consist of students from the first 2 years, who were probably less affected in terms of specialty selection and doing external rotations.

 This study was conducted within 1 year of the COVID pandemic and many of us would probably agree, there has been a significant amount of development, which makes the study less relevant. Yet, one can understand it does take time to process the data and prepare a manuscript (although it has taken over a year to do so in this case).

 I think statistically, the median would be more relevant to the mean as an average. I would suggest the authors providing that as well in case the results are skewed. As well as the average score, it may be useful to include percentages for certain questions, e.g., how many students answered “Somewhat agree” or “Agree” in the “I have considered quitting medical school during the pandemic” question. It will be useful to include that in the supplemental information.

 In addition, I feel the text is rather inconsistent when it comes to the description of data. For example, “When questioned about the adequacy of their medical education regarding epidemics, they felt neutral (3.49 ± 1.08).” and “The students somewhat agreed that… it is their responsibility to volunteer during the pandemic (3.37 ± 1.12)”. A score of 3.49 is described as “neutral” and yet a lower score of 3.37 is described as “somewhat agreed”.

 As the authors pointed out, this is a single-institution study and may therefore not apply to other medical schools. Of note, in other countries, such as in the UK, newly-qualified doctors must rotate through a number of different specialties prior to applying for a specialty-specific residency program. Therefore, the issue in exploring different specialties during medical school becomes less important. It will be useful for the authors to discuss more recommendations on helping medical students, e.g., the introduction of virtual open days and rotations may help to keep them informed on different specialties.

Is the work clearly and accurately presented and does it cite the current literature?

Yes

If applicable, is the statistical analysis and its interpretation appropriate?

Partly

Are all the source data underlying the results available to ensure full reproducibility?

Partly

Is the study design appropriate and is the work technically sound?

Yes

Are the conclusions drawn adequately supported by the results?

Partly

Are sufficient details of methods and analysis provided to allow replication by others?

Yes

Reviewer Expertise:

My main clinical interests are in neurosurgery, particularly neuromodulation. In addition, I am interested in medical education and have completed a postgraduate certificate in medical education. I have also taught, examined and mentored medical students on multiple occasions.

I confirm that I have read this submission and believe that I have an appropriate level of expertise to confirm that it is of an acceptable scientific standard, however I have significant reservations, as outlined above.

# Response

**Published:** 2023-02-16

**Authors:** Heba Mahjoub

**Affiliations:** Johns Hopkins University School of Medicine, USA

**Reviewer 1, Jason Yuen, Department of Neurologic Surgery, Mayo Clinic, Rochester, MN, USA:**

**Approved with Reservations**

**Reviewer's comment:**

 The authors of this paper conducted a single-institution, cross-sectional study of the perspectives of medical students on how the COVID pandemic impacted their education, well-being and future career. In particular, the authors found that, based on answers in the form of Likert scales from an online survey, medical students encountered difficulties in exploring different specialties. Yet, they continued to view medicine as a positive career choice.

 Pros

 How COVID-19 impacts medical education remains highly topical given the evolving nature of the pandemic, although numerous articles on a similar topic have been published for the past 2 years. The questions of the survey were well-designed to capture the numerous ways COVID might affect a medical student.

**Authors response:**

 We thank you for your diligent review and positive reaction to our important work studying students’ perceived impact on their career trajectories during the COVID-19 pandemic.

**Reviewer's comment:**

 Cons

 The study is heavily limited by the low response rate (13%). Therefore, the results may be confounded by selection bias, which the authors have rightly acknowledged.

 Furthermore, a significant proportion of the samples consist of students from the first 2 years, who were probably less affected in terms of specialty selection and doing external rotations.

 This study was conducted within 1 year of the COVID pandemic and many of us would probably agree, there has been a significant amount of development, which makes the study less relevant. Yet, one can understand it does take time to process the data and prepare a manuscript (although it has taken over a year to do so in this case).

**Authors response: **The authors do acknowledge the low response rate, and we attribute survey fatigue as a major contributor given this time period was one where students were inundated with virtual surveys, especially since in-person research activities were shut-down to prevent spread of infection. However, we believe that first and second years were a crucial group to study, as these are the years where many students first encounter or begin to explore their preferred specialty. We also recognize the time since data collection and manuscript preparation, though this was heavily impacted by COVID-19 roadblocks such as illness and delayed reviewer identification.

**Reviewer's comment:**

 I think statistically, the median would be more relevant to the mean as an average. I would suggest the authors providing that as well in case the results are skewed. As well as the average score, it may be useful to include percentages for certain questions, e.g., how many students answered “Somewhat agree” or “Agree” in the “I have considered quitting medical school during the pandemic” question. It will be useful to include that in the supplemental information.

**Authors response: **We checked for normality by analyzing kurtosis and skew using the Jarque-Barre test, and found the majority (16/23) of our questions met the criteria. Because of this, we do believe that the mean results are not skewed, but we have now also included median, 1 ^st^ quartile, and 75 ^th^ quartile in our final table to aid readers in visualizing the results.

**Reviewer's comment:**

 In addition, I feel the text is rather inconsistent when it comes to the description of data. For example, “When questioned about the adequacy of their medical education regarding epidemics, they felt neutral (3.49 ± 1.08).” and “The students somewhat agreed that… it is their responsibility to volunteer during the pandemic (3.37 ± 1.12)”. A score of 3.49 is described as “neutral” and yet a lower score of 3.37 is described as “somewhat agreed”.

**Authors response: **Thank you for bringing this to our attention. We have now added a line in the methods section clarifying which range value constitutes each variable:

*“Any value which rounded to the nearest 0.5 was included in the score above, for example a value of 3.49 was categorized as “somewhat agree”.”*

  We also remedied 2 inconsistencies in the following lines:

*“When questioned about their medical education regarding epidemics, they somewhat agreed that their education was adequate (3.49 ± 1.08).”*

*“The students somewhat agreed that they view the field of medicine more positively since the onset of the COVID-19 pandemic (3.60 ± 1.09), but they felt neutral regarding their responsibility to volunteer during the pandemic (3.37 ± 1.12).”*

**Reviewer's comment:**

 As the authors pointed out, this is a single-institution study and may therefore not apply to other medical schools. Of note, in other countries, such as in the UK, newly-qualified doctors must rotate through a number of different specialties prior to applying for a specialty-specific residency program. Therefore, the issue in exploring different specialties during medical school becomes less important. It will be useful for the authors to discuss more recommendations on helping medical students, e.g., the introduction of virtual open days and rotations may help to keep them informed on different specialties.

**Authors response: **Thank you for this unique perspective. It is true that these findings would be less relevant in other countries, such as the UK. We have included the following recommendation in the discussion section to be more helpful for students from all countries:

*“In the future, students may benefit from earlier exposure to different specialties in a virtual platform. One example would be each specialty creating a “Day in the Life” video at various institutions, which would expose students to the daily routine of each and help them make a decision about which experiences suited them best.”*

    
